# Sub-specialization in plastic surgery in Sub-saharan Africa: capacities, gaps and opportunities

**DOI:** 10.11604/pamj.2014.19.13.4190

**Published:** 2014-09-08

**Authors:** Abdulrasheed Ibrahim

**Affiliations:** 1 Division of Plastic surgery, Department of Surgery, Ahmadu Bello University Teaching Hospital, Zaria, Kaduna state, Nigeria

**Keywords:** sub-specialization, fellowships, plastic surgery, Sub-saharan Africa

## Abstract

The skill set of a plastic surgeon, which addresses a broad range of soft tissue conditions that are prevalent in sub-Saharan Africa, remains relevant in the unmet need for surgical care. Recently, there has being a major paradigm shift from discipline-based to disease-based care, resulting in an emerging component of patient-centered care; adequate access to subspecialty care in plastic and reconstructive surgery. Given the need for an evolution in sub-specialization, this article focuses on the benefits and future role of differentiation of plastic surgeons into sub-specialty training pathways in sub-Saharan Africa.

## Commentary

There is a growing realization of the importance of surgical conditions in the overall burden of disease in low and middle-income countries [1]. The primary indicator of the burden of premature mortality and disability, including temporary disability is the disability-adjusted life year (DALY). DALY is the sum of life years lost due to premature mortality and years lived with disability adjusted for severity [2].

Sixty-six per cent of the estimated DALYs comprising the global burden of disease attributed to surgical conditions are due to injuries, malignancies, and congenital anomalies. These account for an estimated 38 DALYs per 1,000 population in Africa, and commonly require plastic surgical expertise [3–5]. In addition, the World Health Organization has defined an emergency and essential surgical care package that includes 35 procedures [6]. Patients in every population are expected to have easy access to them, and plastic and reconstructive surgeons perform approximately one half of these emergency and essential procedures [6].

Surveys from several countries in sub-Saharan Africa have been published documenting severe deficiencies in surgical capacity [5, 7–10]. Most of the countries have less than one surgeon per 100,000 people. It is also estimated that the availability of surgical services is at least ten times below the minimal needs [11, 12]. Even more glaring is the disconnect between the burden of disease amenable to plastic surgery and the supply of specialty expertise [13]. This disconnect is one of geography, training, financial incentives, and of public policy and implicit societal preferences [5]. Nigeria with a population of over 140 million citizens has only seventy-two (72) plastic surgeons actively practicing in the country [11]. There are three (3) plastic surgeons in Uganda [14] and one (1) in Zambia [5]. This situation is further aggravated by a major paradigm shift from discipline-based to disease-based care, resulting in an emerging component of patient - centered care; adequate access to subspecialty care in plastic and reconstructive surgery [15–17].

### Metrics of sub-specialization in plastic surgery

Sub-specialization is a necessary and logical phenomenon that manifests when the knowledge base, the technical skills and the technology of a given field of study are more than one person can subsume [18]. Perhaps the single greatest factor driving sub-specialization globally, is the astonishing magnitude of the pace of change [16, 17]. Research is constantly presenting new procedures, advancing protocols and most importantly, contributing to a greater understanding of the pathogenesis and management of plastic surgery conditions [18]. It is a positive and evolving process by which each generation is able to provide better patient care and conduct research and training more effectively than the previous generation [19].

The postgraduate residency training program in surgery is affiliated to two Colleges in sub-Saharan Africa. The West African College of Surgeons serves five Anglophone countries in West Africa and the College of Surgeons of East, Central and Southern Africa (COSECSA) offers residency in surgery for 10 member countries. The Colleges’ offer residency in general, pediatric, cardiothoracic, neurosurgery, orthopedic, and plastic surgery as well as in ophthalmology and urology. The colleges identifies a resident as a trainee enrolled in a recognized specialty training program accredited with one of the institutions in the sub region. They are responsible for the curricula, and examination of candidates for registration as consultant surgeons [13]. In contemporary parlance, the training programs for subspecialists are referred to as fellowships [19]. A surgical fellowship is often a 1- or 2-year clinical experience performed after residency (postgraduate) training in plastic and reconstructive surgery. The fellowship training provides a focused, intensive, educational experience in a recognized subspecialty area [19]. A fellowship may be done in Burns, hand and upper extremity, craniofacial, breast, micro vascular, aesthetic and pediatric plastic surgery [20]. The goal of the fellowship training is to produce an expert in a focused area of plastic surgery. In addition, many fellowships train surgeons who can become leaders in the fields of research and surgical education. It is these surgeons who play an important role in teaching medical students, residents and allied health professionals.

The motives for seeking subspecialty training are numerous and varied. Plastic surgeons seek subspecialty training in order to obtain additional expertise in a narrow field so that they can provide superior patient care. Some seek fellowship training in order to reach a sense of mastery in a subspecialty field [19]. Some pursue fellowship training because they find one particular subspecialty more satisfying and wish to limit their practice to that area. On a less altruistic note, subspecialty training may be pursued to achieve a marketing advantage over colleagues who practice the specialty more broadly, or to attain a higher level of job security. Other motives include seeking a higher professional income and searching for a safe harbor in terms of the threat of malpractice litigation [19].

### Bridging the gap

The training of fellows in subspecialties in sub-Saharan Africa has conventionally entailed candidates going to host institutions in the developed world for 12 to 24 months of training and subsequently returning to their home countries to practice their newly acquired skills. However, a significant limitation to this traditional model has been the lack of an enabling environment and/or institutional capacity in the fellow's home country. Such limitations prevent the new fellow from practicing in a meaningful way the new skills he or she has learned, to the disadvantage of both the patients and the fellow. This can result in the fellow electing to license and practice elsewhere if there are no other pathways of career development in place in the fellow's home country [15].

Observerships are also available, in which the trainee can visit a centre with a particular reputation or expertise, to learn by observation rather than practical involvement in patient care. Constraints on salary, time and medical registration make such observerships a valuable experience.

"Blitz surgery" denotes a swift, short surgical campaign that is focused on an area of specific need. Blitz surgery encompasses any such program that lasts 3 weeks or less. Sub-Saharan Africa has for many years offered a fertile ground for surgical blitzes, providing a huge reserve of surgical pathology. Most blitz surgeries are organized and funded by nongovernmental organizations (NGOs) or philanthropic individuals. They are generally reconstructive in nature: the correction of cleft lips and palates, cancrum oris and post burn sequelae [10].

### Opportunities: the sandwich model

To address a rapidly changing epidemiological profile in sub-Saharan Africa, it is crucial to develop fellowship models for sustainable, quality subspecialty services in plastic and reconstructive surgery [15]. The Sandwich fellowship model aims to link the development of the fellow's career with the development of institutional capacity. The unique name of the fellowship model acknowledges the fact that the candidate's training experience occurs in cumulative layers at diverse geographic sites [15]. The program involves the fellow completing a series of rotations spent in both a developed world institution as well as a home-based institution. The home-based institution must demonstrate a commitment to the development of subspecialty areas in plastic surgery and a capacity relevant for the practice of that subspecialty. As compared to a traditional fellowship, it permits some continuity of care while the fellow is at home, thus a higher likelihood of integrating learning acquired into clinical practice [15]. The net effect of these efforts is the potential to increase the production of high-quality plastic surgeons likely to serve populations in their country of training [21].

There are several possible funding sources for fellows participating in a Sandwich fellowship: institutional funding from universities, grant agencies, industry, public-private partnerships, and, potentially, the government of the developing nation. It should be noted that the Sandwich model has a potential cost reduction compared with the traditional model because the fellow′s family may remain at home instead of joining the fellow overseas [15]. This is an important consideration given that a fellowship training program can place a significant burden on the spouse and family, as many studies have shown [22, 23]. Relocating to another country for 1 to 2 years and living on a limited income with extended working hours can be difficult on the family and spouse [24]. With this model, the fellow′s home institution will be responsible for fellow and trainers travel, local trainer accommodation, and the fellow′s tuition, salary, and living expenses even while away. Alternatively the tuition, salary, and living expenses could be assumed by external funding if obtained [15].

Engaging in this type of partnership is mutually beneficial. The program fosters the growth of an international network of plastic surgeons. As fellows graduate and return to their countries, the mentoring and peer relationships they developed during training promotes the development of a plastic surgery community that is not bound by borders or oceans [25]. Equally important, is that the trainers gain from continued professional growth in a challenging developing world setting that allows individuals to encounter several unique pathologies not commonly seen in their local populations [15]. The long-term goal would be to expand the scope of the fellowship to include greater emphasis on teaching and management, allowing the fellow to return home and contribute to the development of the subspecialty. Further development and expansion of the Sandwich fellowship in the same subspecialty area would allow a future fellow to receive guidance from the previous graduate. This model thus ultimately provides a home-based trainer, thereby creating a more supportive teaching environment. Once the hospital sustainably achieves adequate institutional capacity, there is also the promise of creating local or regional fellowship programs that could initially be supported by the overseas partner but, eventually, become fully independent [15].

Sub-specialization is advantageous for the professional and the field that it serves. For the plastic surgeon, it allows for a manageable knowledge base and an attainable technical skill set. Sub-specialization means larger patient volumes per unit with improved training opportunities. This facilitates the establishment of sub-specialist and super-specialist interests. In addition, clinically meaningful trials are easier to perform in specialized environments [26]. For its patient population, adequate subspecialty expertise is vital for sustainable quality services that are appropriate to the local environment while achieving global best practice standards [15, 17, 18, 27].

## Conclusion

Plastic surgery remains relevant to the unmet need for surgery in sub-Saharan Africa. Burns, traumatic injuries, infections, malignancies, and congenital anomalies are estimated to make up half of the world's burden of all surgical diseases. The sandwich model systematically links the professional development of a fellow from a developing world institution with capacity development at the fellow's home institution. This enhances an enabling environment at home that allows both personal and professional growth of the fellow, reducing the risk of brain drain. The beneficiaries of this process are the hospitals, their staff, and most importantly the patients
